# Circ_ZFR contributes to the paclitaxel resistance and progression of non-small cell lung cancer by upregulating KPNA4 through sponging miR-195-5p

**DOI:** 10.1186/s12935-020-01702-0

**Published:** 2021-01-06

**Authors:** Junmin Li, Rongmei Fan, Hui Xiao

**Affiliations:** 1 Department of Respiratory Medicine, Renmin Hospital, Hubei University of Medicine, Shiyan, Hubei China; 2 Department of Critical Care Medicine, Taihe Hospital, Hubei University of Medicine, No. 32 Renmin South Road, Shiyan, 442000 Hubei China

**Keywords:** NSCLC, PTX, Circ_zfr, miR-195-5p, KPNA4

## Abstract

**Background:**

A growing body of evidence has demonstrated the vital roles of circular RNAs (circRNAs) in cancer progression and drug resistance. We intended to explore the roles and mechanisms of circ_ZFR in the paclitaxel (PTX) resistance and progression of non-small cell lung cancer (NSCLC).

**Methods:**

Two NSCLC cell lines A549 and H460 were used in this study. Quantitative real-time polymerase chain reaction (qRT-PCR) assay was conducted to measure the levels of circ_ZFR, ZFR, miR-195-5p and karyopherin subunit alpha 4 (KPNA4) mRNA. RNase R assay was used to analyze the characteristic of circ_ZFR. MTT assay was carried out to assess PTX resistance and cell proliferation. Flow cytometry analysis was utilized to analyze cell cycle and apoptosis. Transwell assay was used to examine cell migration and invasion. Western blot assay was conducted to measure the protein levels of Ki67, Twist1, E-cadherin and KPNA4. Dual-luciferase reporter assay was adopted to verify the combination between miR-195-5p and circ_ZFR or KPNA4. Murine xenograft model assay was used to investigate the effect of circ_ZFR on PTX resistance of NSCLC in vivo.

**Results:**

Circ_ZFR level was enhanced in PTX-resistant NSCLC tissues and cells. Knockdown of circ_ZFR suppressed PTX resistance, cell cycle process, proliferation, migration and invasion and induced apoptosis in PTX-resistant NSCLC cells. For mechanism analysis, circ_ZFR knockdown markedly downregulated the expression of KPNA4 by sponging miR-195-5p, thereby promoting PTX sensitivity and suppressing cell progression in PTX-resistant NSCLC cells. In addition, circ_ZFR silencing enhanced PTX sensitivity of NSCLC in vivo.

**Conclusion:**

Circ_ZFR knockdown played a positive role in overcoming PTX resistance of NSCLC via regulating miR-195-5p/KPNA4 axis, which might provide a possible circRNA-targeted therapy for NSCLC.

## Highlights

1. High expression of circ_ZFR is observed in PTX-resistant NSCLC tissues and cells.
2. Circ_ZFR promotes PTX resistance, cell proliferation, migration and invasion and suppresses cell cycle arrest and apoptosis in PTX-resistant NSCLC cells.
3. Circ_ZFR regulates PTX resistance and cell progression in PTX-resistant NSCLC cells by regulating miR-195-5p/KPNA4 axis.
4. Circ_ZFR enhances PTX resistance of NSCLC in vivo.

## Introduction

Non-small cell lung cancer (NSCLC) is the major type of lung cancer with an occupancy case rate of more than 80% [1, 2]. Although tremendous efforts have been made in the diagnosis and therapy of NSCLC, it remains to be the main cause of cancer-related mortality [3]. Currently, the treatment strategies for NSCLC include surgery, radiotherapy, chemotherapy and immunotherapy [4]. Paclitaxel (PTX) is an antineoplastic drug in various types of tumors, including NSCLC [5]. However, the acquisition of drug resistance is a big obstacle for chemotherapy [6]. Thus, it is crucial to discover new methods to overcome the resistance of NSCLC patients to PTX.

As a novel class of non-coding RNAs (ncRNAs), circular RNAs (circRNAs) possess covalent closed loops and function as crucial regulators in tumor carcinogenesis and chemoresistance [7, 8]. For example, circPVT1 contributed to PTX resistance and cell invasion and repressed apoptosis in gastric cancer [9]. Circ_0035483 improved gemcitabine resistance and facilitated tumorigenesis in renal cancer through regulating miR-335/CCNB1 axis [10]. In NSCLC, several circRNAs, such as circ_0002483 [11], circ_0076305 [12] and circ_0004015 [13], have been demonstrated to be closely related to drug resistance. Moreover, circ_0011292 improved PTX resistance in NSCLC by regulating miR-379-5p/tripartite motif-containing protein 65 (TRIM65) axis [14]. As for circ_ZFR (also termed as circ_0072083), Li et al. [15] claimed that circ_ZFR deficiency could enhance the inhibition of tumor growth induced by cisplatin (DDP) in NSCLC via modulation of miR-545-3p/CBLL1 axis. However, the contributions of circ_ZFR to PTX resistance of NSCLC remain largely unexplored.

MicroRNAs (miRNAs) are ncRNAs (~ 22 nucleotides) that post-transcriptionally alter gene expression through targeting the 3′ untranslated region (3′UTR) of target mRNAs [16, 17]. Presently, miRNAs have been identified to be associated with tumor progression and chemoresistance. Among these, miR-195-5p was verified to be associated with the carcinogenesis and drug resistance of multiple cancers. For example, Dai et al. [18] suggested that miR-195-5p overexpression facilitated the sensitivity of ovarian cancer to DDP by targeting phosphoserine aminotransferase 1 (PSAT1). Feng et al. [19] manifested that miR-195-5p restrained 5-fluoruracil resistance and accelerated apoptosis in chemoresistant colorectal cancer by binding to glycerophosphodiester phosphodiesterase domain containing 5 (GDPD5). In NSCLC, miR-195-5p was also confirmed to be involved in chemosensitivity and carcinogenesis [20, 21]. Karyopherin subunit alpha 4 (KPNA4), a specific KPNA subtype, plays a promotional role in the progression of NSCLC through circCCDC66/miR-33a-5p/KPNA4 regulatory axis [22]. However, the precise roles of KPNA4 in the chemosensitivity of NSCLC are still unclear. Moreover, whether KPNA4 can be targeted by miR-195-5p is not reported.

The indispensable effects of circRNA/miRNA/mRNA regulatory network on tumor progression and chemoresistance have been gradually elucidated [23, 24]. In this research, we found that miR-195-5p possessed the binding sequences of circ_ZFR and KPNA4, indicating that circ_ZFR might function as the sponge for miR-195-5p to elevate KPNA4 expression. Hence, we aimed to investigate the functions of circ_ZFR/miR-195-5p/KPNA4 axis on the carcinogenesis and PTX resistance of NSCLC.

## Materials and methods

### Tissues acquisition

64 NSCLC tissue specimens and 44 adjacent normal tissue specimens were harvested from NSCLC patients at Renmin Hospital, Hubei University of Medicine. According to the response of NSCLC patients to PTX, the samples were divided into 2 groups: treatment-sensitive group (n = 44) and treatment-resistant group (n = 20). The tissue specimens were preserved at − 80 °C until use. The work was approved by the Ethics Committee of Renmin Hospital, Hubei University of Medicine. All patients enrolled in this research provided written informed consents.

### Cell culture

Primary human bronchial epithelial cells (HBE) and NSCLC cells (A549 and H460) were purchased from Procell (CL-0346; CL-0016; CL-0299; Wuhan, China). The corresponding PTX-resistant NSCLC cells (A549/PTX and H460/PTX) were established by exposing the parental cells into escalating doses of PTX (SP8020; Solarbio, Beijing, China). All cells were grown overnight in RPMI1640 medium (A4192301; Invitrogen, Carlsbad, CA, USA) supplemented with 10% fetal bovine serum (16140063, FBS; Invitrogen) and 1% penicillin–streptomycin (10378016; Invitrogen) at 37 °C with 5% CO_2_. In addition, 5 nM PTX (SP8020; Solarbio) was supplemented into the culture medium to maintain the resistance of A549/PTX and H460/PTX cells.

### Quantitative real-time polymerase chain reaction (qRT-PCR)

Total RNA was isolated utilizing Rneasy Mini kit (Cat No./ID: 74104; Qiagen, Valencia, CA, USA) and determined utilizing NanoDrop 2000c spectrophotometer (Thermo Fisher Scientific, Waltham, MA, USA). Next, cDNAs were generated using M-MLV Reverse Transcriptase Kit (M1701; Promega, Madison, WI, USA) or TaqMan MicroRNA Reverse Transcription Kit (4366596; Applied Biosystems, Foster City, CA, USA). Thereafter, qRT-PCR reaction was manipulated on the StepOnePlus Real-Time PCR System (4376598; Applied Biosystems) with SYBR Green PCR Master Mix (4309155; Invitrogen) and specific primers (RIBOBIO, Guangzhou, China). The primers were: circ_ZFR: (F: 5′-AACCACCACAGATTCACTAT-3′ and R: 5′-AACCACCACAGATTCACTAT-3′); ZFR: (F: 5′-TCCCAATGCTAAGGAGATGC-3′ and R: 5′-TTCTTCTCGTCTTCGCCAGT-3′); miR-195-5p: (F: 5′-CTGGAGCAGCACAGCCAATA-3′ and R: 5′- AGCTTCCCTGGCTCTAGCA-3′); KPNA4: (F: 5′-CAGGAGATTCTTCCAGCCCTTTGTGT-3′ and R: 5′-ATTACCATCTGTATTTGTTCATTGCCAGCATC-3′); glyceraldehyde 3-phosphate dehydrogenase (GAPDH): (F: 5′-TATGATGATATCAAGAGGGTAGT-3′ and R: 5′-TGTATCCAAACTCATTGTCATAC-3′); U6: (F: 5′-CTCGCTTCGGCAGCACA-3′ and R: 5′-AACGCTTCACGAATTTGCGT-3′). The relative expression was calculated using the 2^−ΔΔCq^ method [25]. GAPDH and U6 were used as internal references.

### RNase R assay

To verify the loop structure of circ_ZFR, total RNA (10 μg) from A549 and H460 cells was treated with or without RNase R (20 mg/mL; RNR07250; Epicenter Biotechnologies, Madison, WI, USA) for 15 min at 37 °C. The abundance of circ_ZFR and linear ZFR mRNA was examined through qRT-PCR analysis.

### Subcellular fraction assay

The nuclear and cytoplasmic fractions of A549 and H460 cells were separated with PARIS Kit (AM1921; Invitrogen) in line with the protocols of manufacturer. Thereafter, the levels of GAPDH, U6 and circ_ZFR were determined using qRT-PCR analysis. U6 and GAPDH were employed as the controls of nuclear transcript and cytoplasmic transcript, respectively.

### Cell transfection

Circ_ZFR small interfering RNA (si-circ_ZFR; 5′-CAAATTTATGCCCAGCCGGCT-3′) and si-NC (si-NC, 5′-UUCUCCGAACGUGUCACUTT-3′), miR-195-5p mimics (miR-195-5p; 5′-UAGCAGCACAGAAAUAUUGGC-3′) and miR-NC (5′-CGAUCGCAUCAGCAUCGAUUGC-3′), miR-195-5p inhibitors (anti-miR-195-5p; 5′-GCCAAUAUUUCUGUGCUGCUA-3′) and anti-miR-NC (5′-CUAACGCAUGCACAGUCGUACG-3′), the overexpression vector of KPNA4 (KPNA4) and empty pcDNA, circ_ZFR short hairpin RNA (sh-circ_ZFR; 5′-TCAAATTTATGCCCAGCCGGC-3′) and sh-NC (5′-TTCTCCGAACGTGTCACGT-3′) were synthesized by RIBOBIO. Then A549/PTX and H460/PTX cells (2 × 10^4^ cells/well) were plated into 6-well plates for 24 h and then transfected with relevant synthetic oligonucleotides (50 nM) or vectors (2 μg) using Lipofectamine 2000 (11668500; Invitrogen) according to the manufacturers’ instructions.

### 3-(4, 5-dimethyl-2-thiazolyl)-2, 5-diphenyl-2-H-tetrazolium bromide (MTT) assay

PTX resistance and cell proliferation were assessed by MTT assay. To evaluate PTX resistance, the transfected A549/PTX and H460/PTX cells were sowed into 96-well plates (1 × 10^4^ cells/well) and cultivated overnight prior to exposure to different concentrations of PTX (0, 10, 20, 40, 80 and 160 nM; SP8020; Solarbio) for 48 h. Next, 20 μL MTT (5 mg/mL; M1020; Solarbio) was added into each well with incubation for another 4 h at room temperature. Thereafter, 150 µL dimethyl sulfoxide (DMSO; D8371; Solarbio) was added to dissolve the formazan crystals followed by the detection of absorption at 490 nm with a microplate reader (Potenov, Beijing, China). The concentration of PTX causing 50% inhibition of growth (IC_50_) was determined to evaluate PTX resistance.

To analyze cell proliferation, the transfected A549/PTX and H460/PTX cells were sowed into 96-well plates and maintained for 24 h. Then MTT solution (M1020; Solarbio) was added into the well at 0 days, 1 days, 2 days and 3 days with incubation for an additional 4 h. The absorption at 490 nm was examined.

### Flow cytometry analysis

For cell cycle detection, the transfected A549/PTX and H460/PTX cells were fixed overnight with 70% ethanol and then incubated with RNase A (R1030; Solarbio) for 1 h at 37 °C to remove the RNA. Next, the cells were dyed with propidium iodide (C1062S; PI; Beyotime, Shanghai, China). At last, FACScan® flow cytometry (BD Biosciences, San Jose, CA, USA) was adopted to analyze cell proportion at different cell cycle phases.

For cell apoptosis analysis, the transfected cells were collected and suspended in binding buffer followed by staining with Annexin V-fluorescein isothiocyanate (C1062S; FITC) and PI (C1062S) for 15 min in the dark. FACScan® flow cytometry (BD Biosciences) was used to analyze cell apoptosis.

### Transwell assay

The transwell chambers (3374; Corning Incorporated, Corning, NY, USA) coated with or without Matrigel (M8370; Solarbio) were employed to evaluate cell invasion and migration, respectively. In brief, 1.0 × 10^4^ transfected A549/PTX and H460/PTX cells were suspended in serum-free medium and then added into the top compartment of the chambers. The lower compartment was filled with complete culture medium. 24 h later, the migrated and invaded cells were dyed with crystal violet (C8470; Solarbio) and determined using an inverted microscope at the magnification of 100×.

### Western blot assay

Protein extraction was executed utilizing RIPA (P0013C; Beyotime) and protein concentration was examined with NanoDrop 2000c spectrophotometer (Thermo Fisher Scientific). An equal amount of proteins was separated by 10% sodium dodecyl sulfonate-polyacrylamide gel (P1200; Solarbio) electrophoresis and subsequently blotted onto polyvinylidene difluoride membranes (ISEQ00010; Millipore, Billerica, MA, USA). Thereafter, the membranes were blocked in skim milk for 1 h and cultivated with primary antibodies overnight at 4 °C followed by incubation with secondary antibody (bs-40296G-HRP; Bioss, Beijing, China) at indoor temperature for 1 h. The signal of the bands was visualized by the ECL kit (E411-04; Vazyme, Nanjing, China). The primary antibodies used in this study included Ki67 (bs-23103R; Bioss), Twist1 (bs-2441R; Bioss), E-cadherin (bs-1519R; Bioss), N-cadherin (bs20623R; Bioss) KPNA4 (bs-16804R; Bioss), CyclinD1 (bs-0623R; Bioss), Bcl-2 (bs-33411R; Bioss), Bax (bs-0127R; Bioss) and GAPDH (bs-10900R; Bioss).

### Dual-luciferase reporter assay

The relationships among circZFR, miR-195-5p and KPNA4 were predicted by starbase 3.0 and then verified by dual-luciferease reporter assay. In brief, the fragments of circ_ZFR (or KPNA4 3′UTR) including the putative wild-type (wt) or mutant (mut) miR-195-5p binding sites were amplified and cloned into pmirGLO plasmid (E1330; Promega) to form recombinant plasmids circ_ZFR-wt, KPNA4-wt, circ_ZFR-mut and KPNA4-mut. Then A549/PTX and H460/PTX cells were seeded into 12-well plates (5.0 × 10^4^ cells/well) and transfected with recombinant vector (100 ng) together with miR-195-5p or miR-NC (50 nM) using Lipofectamine 2000 (11668500; Invitrogen). After 48 h of co-transfection, the luciferase activity was measured using Dual-Luciferase Reporter Assay System (E1910; Promega). Renilla luciferase activity was normalized to firefly luciferase activity.

### Murine xenograft model

The 4–6 weeks old BALB/c nude mice were bought from Vital River Laboratory (Beijing, China) and divided into 2 groups (n = 7/group). A total of 2 × 10^6^ A549/PTX cells were suspended in PBS and then inoculated into the nude mice. After one week, the mice were administrated with 3 mg/kg PTX (SP8020; Solarbio) or an equal volume of PBS every week for 4 cycles. Tumor volume was examined every week and computed via the equation: (length × width^2^)/2. 4 weeks later, the mice were euthanized. The neoplasms were excised from the mice, weighted and then stored at − 80 °C until use. The animal research was approved by the Ethics Committee of Animal Research of Renmin Hospital, Hubei University of Medicine.

### Statistical analysis

The experiments were executed in triplicate. The results were analyzed with GraphPad Prism 7 and exhibited as mean ± standard deviation (SD). The difference was analyzed using Student’s *t*-test or one-way analysis of variance (ANOVA). The correlation between miR-195-5p and circ_ZFR or KPNA4 was estimated by spearman’s correlation coefficient analysis. It was considered as significant if *P* value less than 0.05.

## Results

### Circ_ZFR was highly expressed in PTX-resistant NSCLC tissues and cell lines

**Fig. 1:**
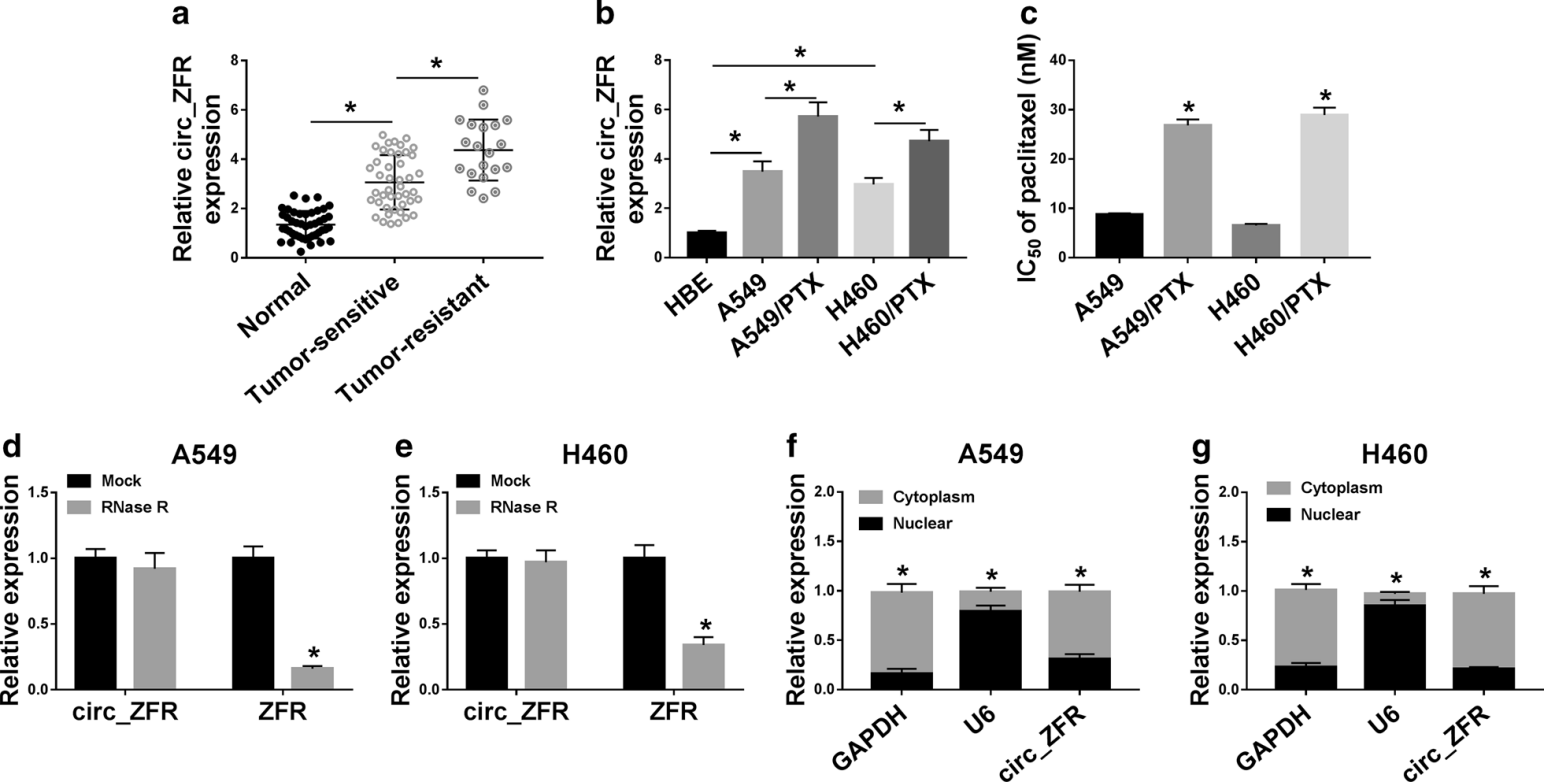
High level of circ_ZFR was observed in PTX-resistant NSCLC tissues and cells. **a** The expression level of circ_ZFR in normal tissues, PTX-sensitive and PTX-resistant NSCLC tissues was determined by qRT-PCR assay. **b** The expression of circ_ZFR in HBE, A549, H460, A549/PTX and H460/PTX cells was examined by qRT-PCR assay. **c** IC_50_ of PTX in A549/PTX and H460/PTX cells was measured by MTT assay. **d**, **e** After total RNA from A549 and H460 cells was treated with or without RNase R, qRT-PCR assay was performed for the levels of circ_ZFR and ZFR. **f**, **g** The expression levels of circ_ZFR and ZFR in the nuclear and cytosolic fractions of A549 and H460 cells were measured by qRT-PCR assay. *P < 0.05

To begin with, qRT-PCR assay was conducted to determine the expression level of circ_ZFR in PTX-resistant and PTX-sensitive NSCLC tissues. As a result, there was an obvious increasing trend in circ_ZFR expression level from normal tissues to PTX-sensitive NSCLC tissues and then to PTX-resistant NSCLC tissues (Fig. 1a). Moreover, we found that circ_ZFR level in A549 and H460 cells was higher than in HBE cells and lower than in A549/PTX and H460/PTX cells (Fig. 1b). Then we evaluated the resistance of A549/PTX and H460/PTX cells to PTX using MTT assay. Our results showed that PTX resistance was produced in A549/PTX and H460/PTX cells, as demonstrated by the elevated IC_50_ value of PTX in A549/PTX and H460/PTX cells (Fig. 1c). Subsequently, the stability of circ_ZFR in A549 and H460 cells was examined by RNase R assay. The results showed that circ_ZFR was resistant to RNase R digestion, while linear ZFR was markedly decreased following the treatment of RNase R (Fig. 1d, e). In addition, we observed that circ_ZFR was mainly enriched in the cytoplasm of A549 and H460 cells (Fig. 1f, g). These results indicated that the dysregulation of circ_ZFR might be involved in the carcinogenesis and chemoresistance of NSCLC.

### Circ_ZFR knockdown suppressed PTX resistance, cell proliferation, migration and invasion and promoted cell cycle arrest and apoptosis in PTX-resistant NSCLC cells

**Fig. 2:**
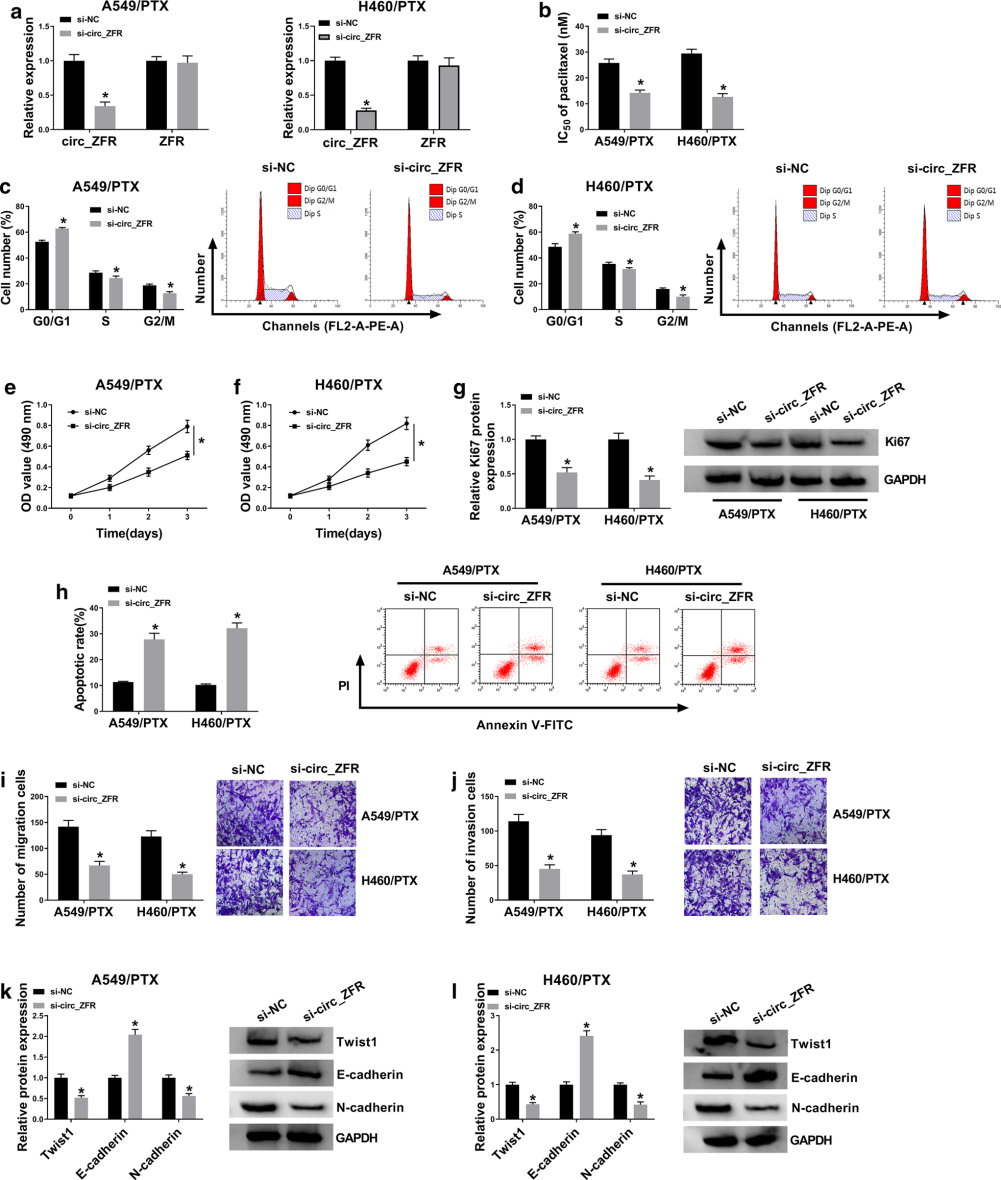
Silencing of circ_ZFR inhibited PTX resistance and malignant cell behaviors in PTX-resistant NSCLC cells. Si-circ_ZFR or si-NC was transfected into A549/PTX and H460/PTX cells. **a** QRT-PCR assay was conducted for circ_ZFR and ZFR expression levels in A549/PTX and H460/PTX cells. **b** MTT assay was adopted to determine IC_50_ of PTX in A549/PTX and H460/PTX cells. **c**, **d** Flow cytometry analysis was performed to analyze cell cycle process in A549/PTX and H460/PTX cells. **e**, **f** MTT assay was utilized for the detection of cell proliferation in A549/PTX and H460/PTX cells. **g** Western blot assay was conducted for Ki67 protein level in A549/PTX and H460/PTX cells. **h** Flow cytometry analysis was used for cell apoptosis ability in A549/PTX and H460/PTX cells. **i**, **j** Transwell assay was conducted to examine the migration and invasion of A549/PTX and H460/PTX cells. **k**, **l** Western blot assay was conducted for the protein levels of Twist1, E-cadherin and N-cadherin in A549/PTX and H460/PTX cells. *P < 0.05

To explore the functions of circ_ZFR in PTX resistance of NSCLC, loss-of-function experiments were carried out by transfecting si-circ_ZFR into A549/PTX and H460/PTX cells to knock down the expression of circ_ZFR. QRT-PCR assay showed that si-circ_ZFR transfection led to a remarkable reduction in circ_ZFR expression in A549/PTX and H460/PTX cells compared to si-NC groups, while the level of ZFR was not changed by si-circ_ZFR transfection (Fig. 2a). MTT assay indicated that IC_50_ of PTX was reduced in A549/PTX and H460/PTX cells transfected with si-circ_ZFR, suggesting that circ_ZFR knockdown repressed the resistance of A549/PTX and H460/PTX cells to PTX (Fig. 2b). As demonstrated by flow cytometry analysis, the numbers of A549/PTX and H460/PTX cells were increased in G0/G1 phase and decreased in S phase following the knockdown of circ_ZFR, indicating cell cycle process was arrested (Fig. 2c, d). Our results also exhibited that circ_ZFR knockdown decreased the level of cell cycle regulatory protein CyclinD1 in A549/PTX and H460/PTX cells relative to si-NC control groups (Additional file 1: Figure S1A). MTT assay showed that compared to control groups, circ_ZFR interference conspicuously inhibited the proliferation of A549/PTX and H460/PTX cells (Fig. 2e, f). Moreover, we found that the level of proliferation-related protein Ki67 was notably downregulated in A549/PTX and H460/PTX cells after circ_ZFR knockdown (Fig. 2g). As illustrated by flow cytomtery analysis, silencing of circ_ZFR evidently enhanced the apoptosis ability of A549/PTX and H460/PTX cells compared to si-NC groups (Fig. 2h). Of note, we detected the effect of circ_ZFR on the expression of apoptotic proteins (Bcl-2 and Bax) in A549/PTX and H460/PTX cells and found that circ_ZFR silencing reduced Bcl-2 level and elevated Bax level in A549/PTX and H460/PTX cells after circ_ZFR deficiency (Additional file 1: Figure S1B, C). The results of transwell assay exhibited that circ_ZFR deficiency drastically inhibited the migration and invasion capacities of A549/PTX and H460/PTX cells compared to control groups (Fig. 2i, j). Additionally, western blot assay was conducted to measure the levels of metastasis-related proteins (Twist1, E-cadherin and N-cadherin) in si-circ_ZFR transfected cells, showing that circ_ZFR knockdown decreased Twist1 and N-cadherin levels and increased E-cadherin level in A549/PTX and H460/PTX cells compared to control groups (Fig. 2k, l). Collectively, circ_ZFR knockdown improved PTX sensitivity and slowed cell progression in PTX-resistant NSCLC cells.

### Circ_ZFR functioned as the sponge of miR-195-5p

**Fig. 3:**
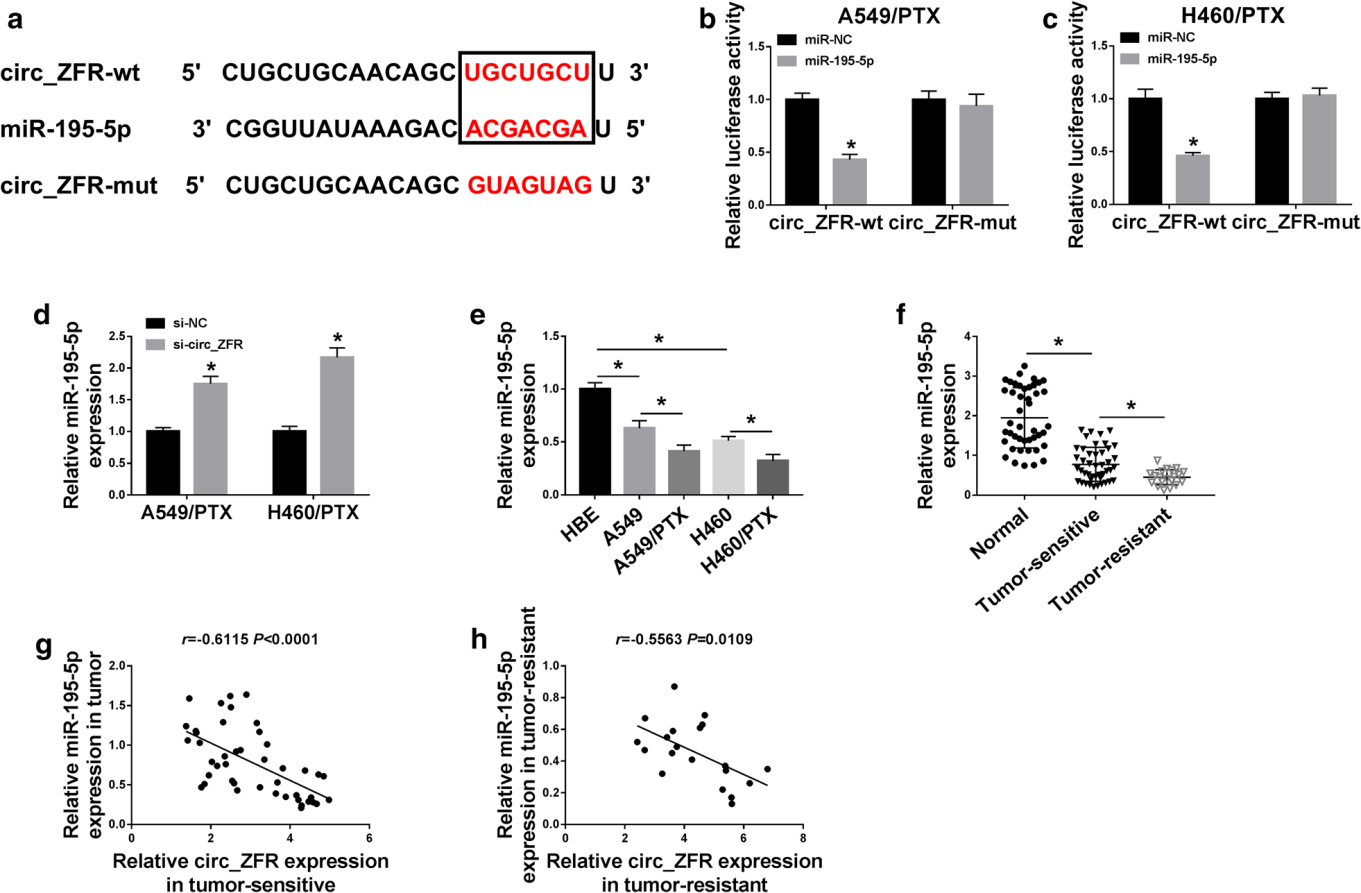
Circ_ZFR sponged miR-195-5p to negatively regulate miR-195-5p expression. **a** The potential binding sites between circ_ZFR and miR-195-5p were shown. **b**, **c** The interaction between circ_ZFR and miR-195-5p in A549/PTX and H460/PTX cells was determined by dual-luciferase reporter assay. **d** The expression level of miR-195-5p in si-NC or si-circ_ZFR transfected A549/PTX and H460/PTX cells was examined by qRT-PCR analysis. **e** QRT-PCR assay was used to determine miR-195-5p level in HBE, A549, H460, A549/PTX and H460/PTX cells. **f** QRT-PCR assay was used for miR-195-5p level in normal tissues, PTX-sensitive and PTX-resistant NSCLC tissues. **g**, **h** The correlation between circ_ZFR and miR-195-5p in PTX-sensitive and PTX-resistant tumor tissues was estimated by spearman’s correlation coefficient analysis. *P < 0.05

To investigate the underlying mechanism of circ_ZFR in regulating PTX resistance and cell progression in PTX-resistant NSCLC cells, online tool starbase 3.0 was used to analyze the potential target of circ_ZFR. As shown in Fig. 3a, miR-195-5p possessed the binding sites of circ_ZFR, indicating that miR-195-5p might be a target of circ_ZFR. To verify it, dual-luciferase reporter assay was conducted. The results exhibited that miR-195-5p transfection markedly inhibited the luciferase activity of circ_ZFR-wt, but did not affect the luciferase activity of circ_ZFR-mut in A549/PTX and H460/PTX cells, further confirming the combination between circ_ZFR and miR-195-5p (Fig. 3b, c). Moreover, we found that circ_ZFR knockdown apparently elevated the expression of miR-195-5p in A549/PTX and H460/PTX cells (Fig. 3d). As expected, miR-195-5p expression in A549 and H460 cells was lower than in HBE cells and higher than in A549/PTX and H460/PTX cells (Fig. 3e). Moreover, there was a decreasing trend in miR-195-5p level from normal tissues to PTX-sensitive NSCLC tissues and then to PTX-resistant NSCLC tissues (Fig. 3f). In addition, we observed that miR-195-5p expression was negatively correlated with circ_ZFR expression in both PTX-sensitive and PTX-resistant tumor tissues (Fig. 3g, h). Taken together, circ_ZFR negatively regulated miR-195-5p expression by directly targeting miR-195-5p.

### Inhibition of miR-195-5p ameliorated the effects of circ_ZFR knockdown on PTX sensitivity and cell progression in PTX-resistant NSCLC cells

**Fig. 4:**
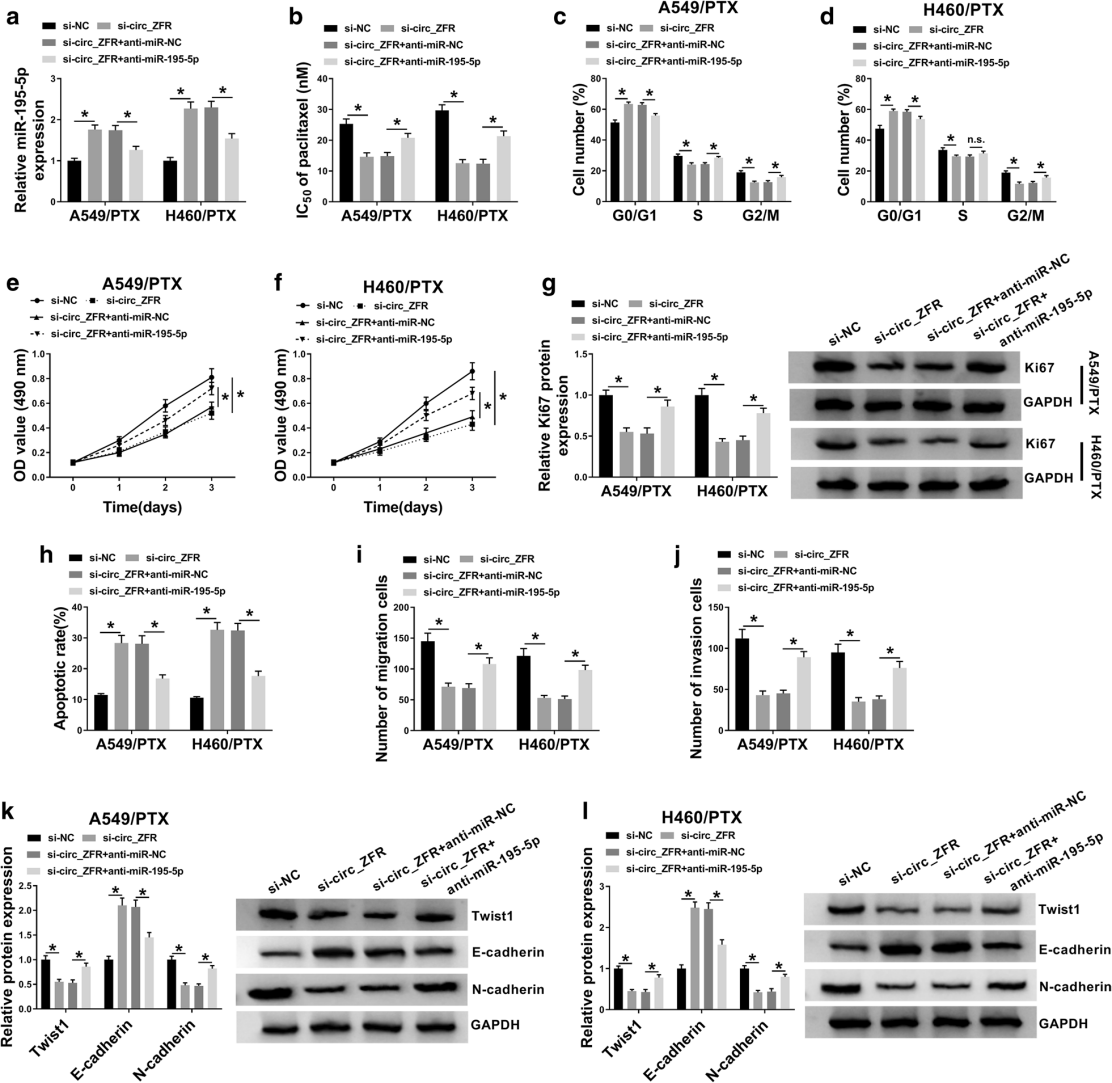
Circ_ZFR promoted PTX resistance and cell progression in PTX-resistant NSCLC cells by targeting miR-195-5p. A549/PTX and H460/PTX cells were treated with si-NC, si-circ_ZFR, si-circ_ZFR + anti-miR-NC or si-circ_ZFR + anti-miR-195-5p. **a** QRT-PCR assay was utilized for miR-195-5p level in A549/PTX and H460/PTX cells. **b** IC_50_ of PTX in A549/PTX and H460/PTX cells was estimated by MTT assay. **c**–**f** Cell cycle and cell proliferation in A549/PTX and H460/PTX cells were analyzed by flow cytometry analysis and MTT assay, respectively. **g** Western blot assay was conducted for Ki67 protein level in A549/PTX and H460/PTX cells. **h** Cell apoptosis, **i**, **j** migration and invasion in A549/PTX and H460/PTX cells were assessed by flow cytometry analysis and transwell assay, respectively. **k**, **l** The protein levels of Twist1, E-cadherin and N-cadherin in A549/PTX and H460/PTX cells were measured through western blot assay. *P < 0.05

Based on the above results, we further investigated whether circ_ZFR could regulate PTX resistance and cell progression by targeting miR-195-5p through rescue experiments. As exhibited in Fig. 4a, anti-miR-195-5p transfection reversed si-circ_ZFR-induced upregulation of miR-195-5p in both A549/PTX and H460/PTX cells. The inhibitory effects of circ_ZFR knockdown on PTX resistance, cell cycle process and proliferation in A549/PTX and H460/PTX cells were all abrogated by decreasing miR-195-5p (Fig. 4b–f). Western blot assay showed that circ_ZFR deficiency decreased the protein level of Ki67 in A549/PTX and H460/PTX cells, while miR-195-5p suppression partially overturned the impact (Fig. 4g). As demonstrated by flow cytometry analysis and transwell assay, miR-195-5p inhibition effectively abated the promotional role on cell apoptosis and the suppressive roles in cell migration and invasion in A549/PTX and H460/PTX cells caused by circ_ZFR knockdown (Fig. 4h–j). In addition, the effects of circ_ZFR deficiency on Twist1, E-cadherin, N-cadherin, CyclinD1, Bcl-2 and Bax levels in A549/PTX and H460/PTX cells were abrogated by downregulating miR-195-5p (Fig. 4k, l, Additional file 1: Figure S1D–F). These outcomes suggested that circ_ZFR knockdown restrained PTX resistance and malignant phenotypes of PTX-resistant NSCLC cells by sponging miR-195-5p.

### KPNA4 was a direct target gene of miR-195-5p

**Fig. 5:**
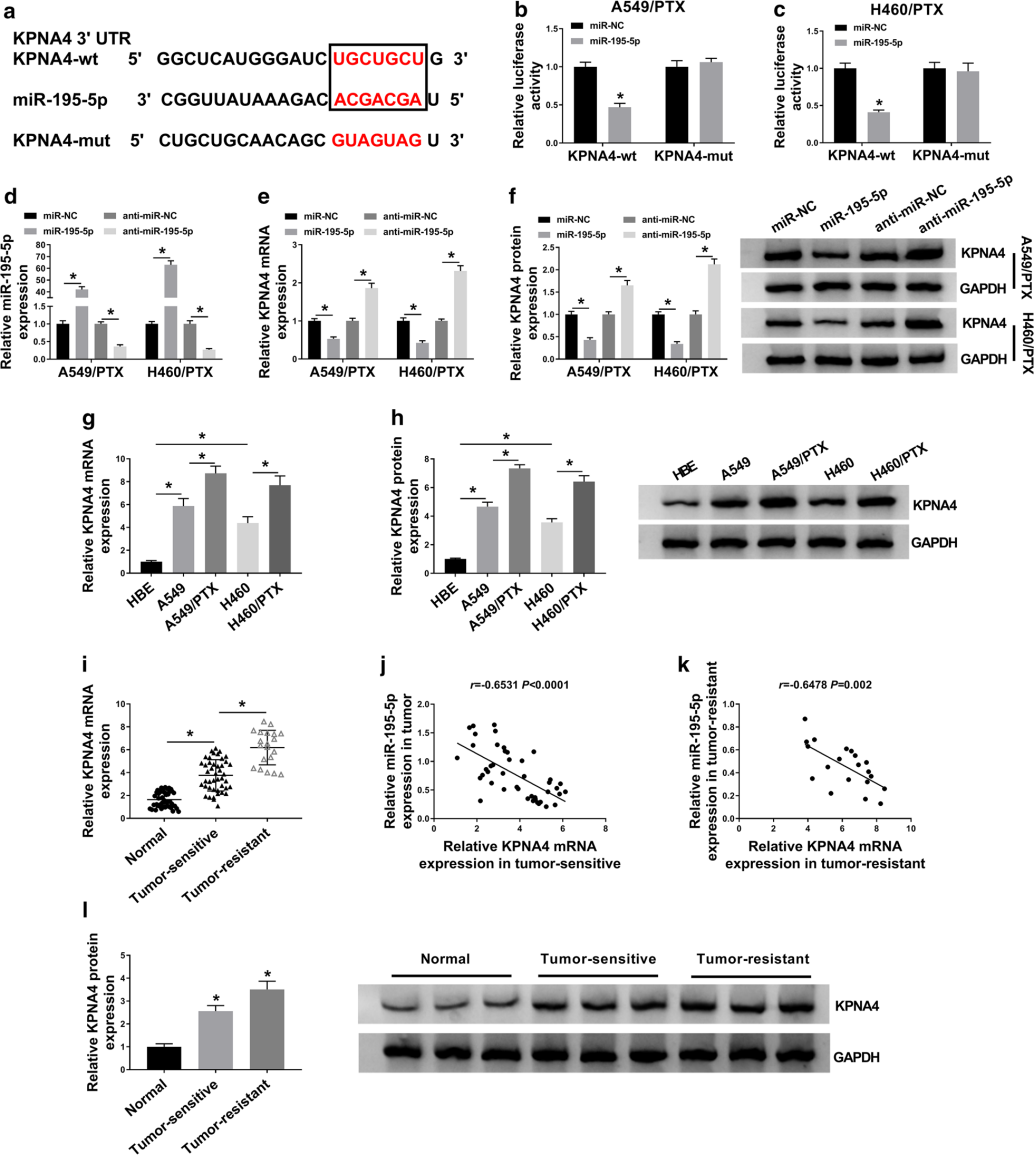
KPNA4 was target gene of miR-195-5p. **a** The predicted binding sites between KPNA4 3′ UTR and miR-195-5p. **b**, **c** The luciferase activity in A549/PTX and H460/PTX cells co-transfected with miR-195-5p/miR-NC and KPNA4-wt/KPNA4-mut was measured by dual-luciferase reporter assay. **d**–**f** The levels of miR-195-5p, KPNA4 mRNA and KPNA4 protein in A549/PTX and H460/PTX cells transfected with miR-195-5p, miR-NC, anti-miR-195-5p or anti-miR-NC were measured by qRT-PCR assay or western blot assay. **g**, **h** The mRNA and protein levels of KPNA4 in HBE, A549, H460, A549/PTX and H460/PTX cells were determined using qRT-PCR assay and western blot assay, respectively. **i** The mRNA level of KPNA4 in normal tissues, PTX-sensitive and PTX-resistant NSCLC tissues was determined by qRT-PCR assay. **j**, **k** The correlation between miR-195-5p and KPNA4 mRNA in PTX-sensitive and PTX-resistant tumor tissues was analyzed by spearman’s correlation coefficient analysis. **l** The protein level of KPNA4 in normal tissues, PTX-sensitive and PTX-resistant NSCLC tissues was measured using western blot assay. *P < 0.05

Through analyzing starbase 3.0, KPNA4 was found to be a target gene of miR-195-5p and their potential binding sites were exhibited in Fig. 5a. Then dual-luciferase reporter assay was carried out to confirm the interaction between miR-195-5p and KPNA4. The results showed that the luciferase activity was apparently inhibited in miR-195-5p and KPNA4-wt co-transfected A549/PTX and H460/PTX cells compared to miR-NC and KPNA4-wt co-transfected groups, whereas no change was observed in KPNA4-mut groups (Fig. 5b, c). Then we transfected miR-195-5p, anti-miR-195-5p or their controls to explore the impact of miR-195-5p on KPNA4 expression. As presented in Fig. 5d, miR-195-5p and anti-miR-195-5p were successfully transfected into A549/PTX and H460/PTX cells. Furthermore, we found that miR-195-5p overexpression markedly reduced the mRNA and protein levels of KPNA4 in A549/PTX and H460/PTX cells, while miR-195-5p inhibition exhibited the opposite results (Fig. 5e, f). In addition, our results showed that the mRNA and protein levels of KPNA4 in A549 and H460 cells were higher than in HBE cells and lower than in A549/PTX and H460/PTX cells (Fig. 5g, h). There was an increasing trend in KPNA4 mRNA and protein levels from normal tissues to PTX-sensitive tumor tissues and then to PTX-resistant tumor tissues (Fig. 5i, l). Through spearman’s correlation coefficient analysis, KPNA4 mRNA level was found to be inversely correlated with miR-195-5p expression in both PTX-resistant and PTX-sensitive NSCLC tissues (Fig. 5j, k). To sum up, miR-195-5p negatively modulated KPNA4 expression by direct interaction.

### Overexpression of miR-195-5p enhanced PTX sensitivity and repressed cell progression in PTX-resistant NSCLC cells by targeting KPNA4

**Fig. 6:**
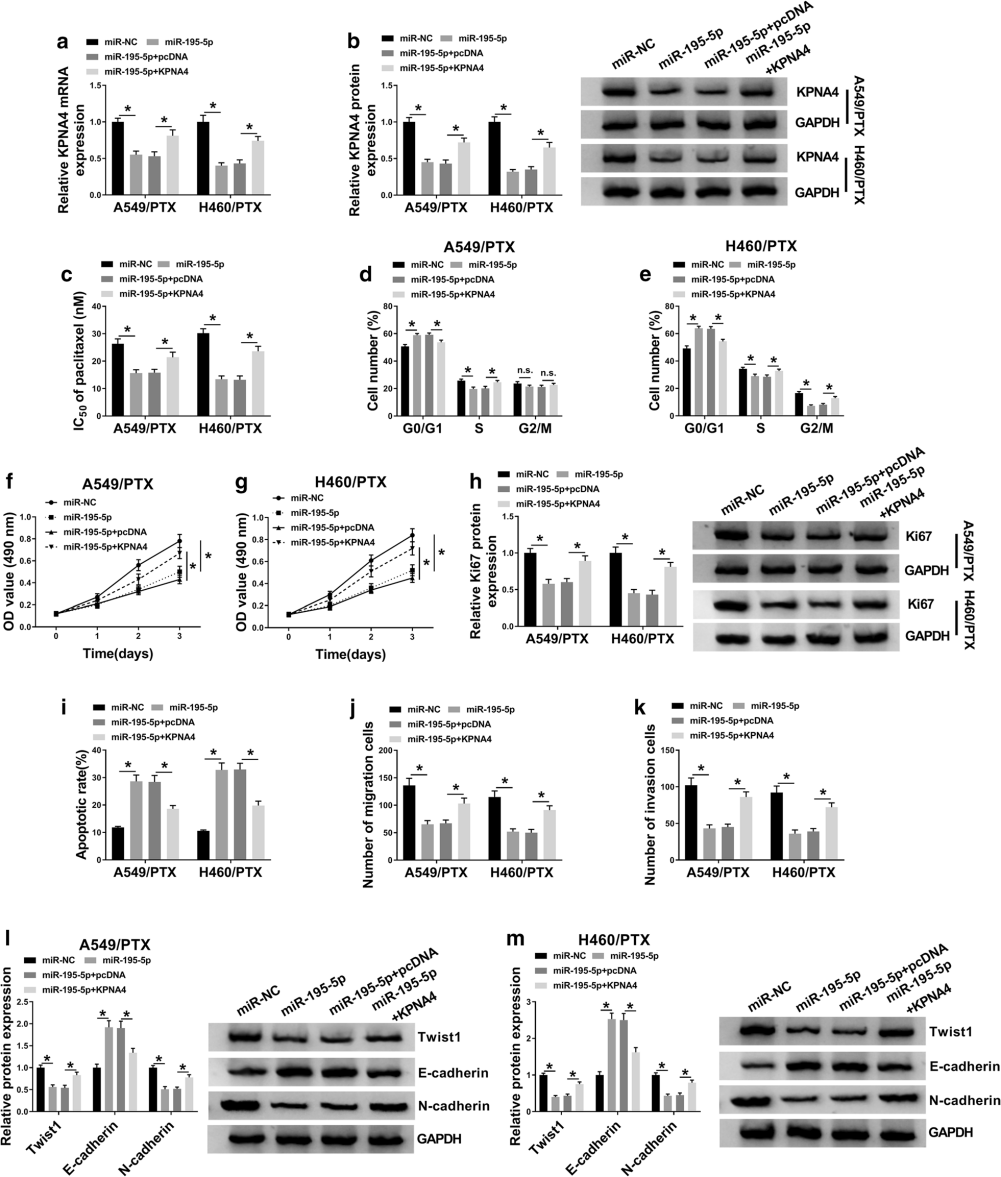
MiR-195-5p overexpression suppressed PTX resistance and malignant behaviors of PTX-resistant NSCLC cells by binding to KPNA4. A549/PTX and H460/PTX cells were assigned to miR-195-5p, miR-NC, miR-195-5p + KPNA4 and miR-195-5p + pcDNA groups. **a**, **b** The mRNA and protein levels of KPNA4 in A549/PTX and H460/PTX cells were determined by qRT-PCR assay and western blot assay, respectively. **c** IC_50_ of PTX in A549/PTX and H460/PTX cells was evaluated using MTT assay. **d**–**g** Cell cycle and cell proliferation in A549/PTX and H460/PTX cells were estimated using flow cytometry analysis and MTT assay, respectively. **h** The protein level of Ki67 in A549/PTX and H460/PTX cells was measured using western blot assay. **i** Cell apoptosis, **j**, **k** migration and invasion in A549/PTX and H460/PTX cells were analyzed by flow cytometry analysis and transwell assay, respectively. **l**, **m** The protein levels of Twist1, E-cadherin and N-cadherin in A549/PTX and H460/PTX cells were measured by western blot assay. *P < 0.05

To explore the roles of miR-195-5p and KPNA4 in PTX resistance and cell progression in PTX-resistant NSCLC cells, A549/PTX and H460/PTX cells were transfected with miR-195-5p, miR-NC, miR-195-5p + KPNA4 or miR-195-5p + pcDNA. As presented in Fig. 6a, b, miR-195-5p transfection obviously reduced the mRNA and protein levels of KPNA4 in A549/PTX and H460/PTX cells, while KPNA4 transfection restored the effects. Through functional experiments, we found that miR-195-5p overexpression distinctly suppressed PTX resistance, cell cycle process and cell proliferation in A549/PTX and H460/PTX cells, while the elevation of KPNA4 effectively reversed the effects (Fig. 6c–g). Western blot assay indicated that miR-195-5p overexpression downregulated the level of Ki67 in A549/PTX and H460/PTX cells, while KPNA4 elevation abolished this impact (Fig. 6h). As suggested by flow cytometry analysis and transwell assay, overexpression of miR-195-5p facilitated cell apoptosis and inhibited cell migration and invasion in A549/PTX and H460/PTX cells, while the effects were rescued by increasing KPNA4 (Fig. 6i–k). In addition, we found that the protein levels of Twist1, N-cadherin, CyclinD1 and Bcl-2 were reduced and the protein levels of E-cadherin and Bax were enhanced in A549/PTX and H460/PTX cells following miR-195-5p overexpression, while the upregulation of KPNA4 attenuated the impacts (Fig. 6l, m, Additional file 1: Figure S1G–I). In a word, miR-195-5p overexpression contributed to PTX sensitivity and impeded cell progression in PTX-resistant NSCLC cells by targeting KPNA4.

### Circ_ZFR knockdown decreased KPNA4 expression through sponging miR-195-5p

**Fig. 7:**
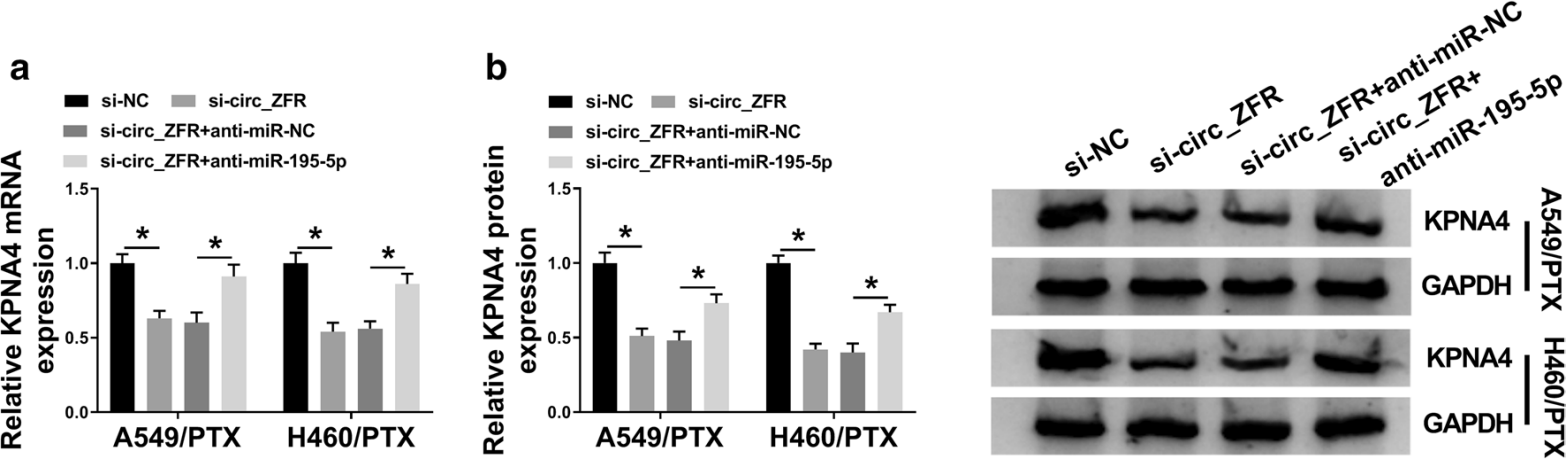
Circ_ZFR silencing downregulated KPNA4 expression by targeting miR-195-5p. **a**, **b** The mRNA and protein levels of KPNA4 in A549/PTX and H460/PTX cells transfected with si-NC, si-circ_ZFR, si-circ_ZFR + anti-miR-NC or si-circ_ZFR + anti-miR-195-5p were measured by qRT-PCR assay and western blot assay, respectively. *P < 0.05

Subsequently, A549/PTX and H460/PTX cells were transfected with si-NC, si-circ_ZFR, si-circ_ZFR + anti-miR-NC or si-circ_ZFR + anti-miR-195-5p to further analyze the associations among circ_ZFR, miR-195-5p and KPNA4. As shown in Fig. 7a, b, silencing of circ_ZFR markedly decreased the mRNA and protein levels of KPNA4 in A549/PTX and H460/PTX cells, while miR-195-5p inhibition effectively restored the effects. Moreover, it was found that circ_ZFR knockdown reduced the mRNA and protein levels of KPNA4 in A549/PTX and H460/PTX cells, while these effects were abolished by KPNA4 overexpression (Additional file 2: Figure S2A, B). Thus, we concluded that circ_ZFR positively regulated KPNA4 expression by sponging miR-195-5p in PTX-resistant NSCLC cells.

### *Circ_ZFR improved PTX resistance of NSCLC *in vivo

**Fig. 8:**
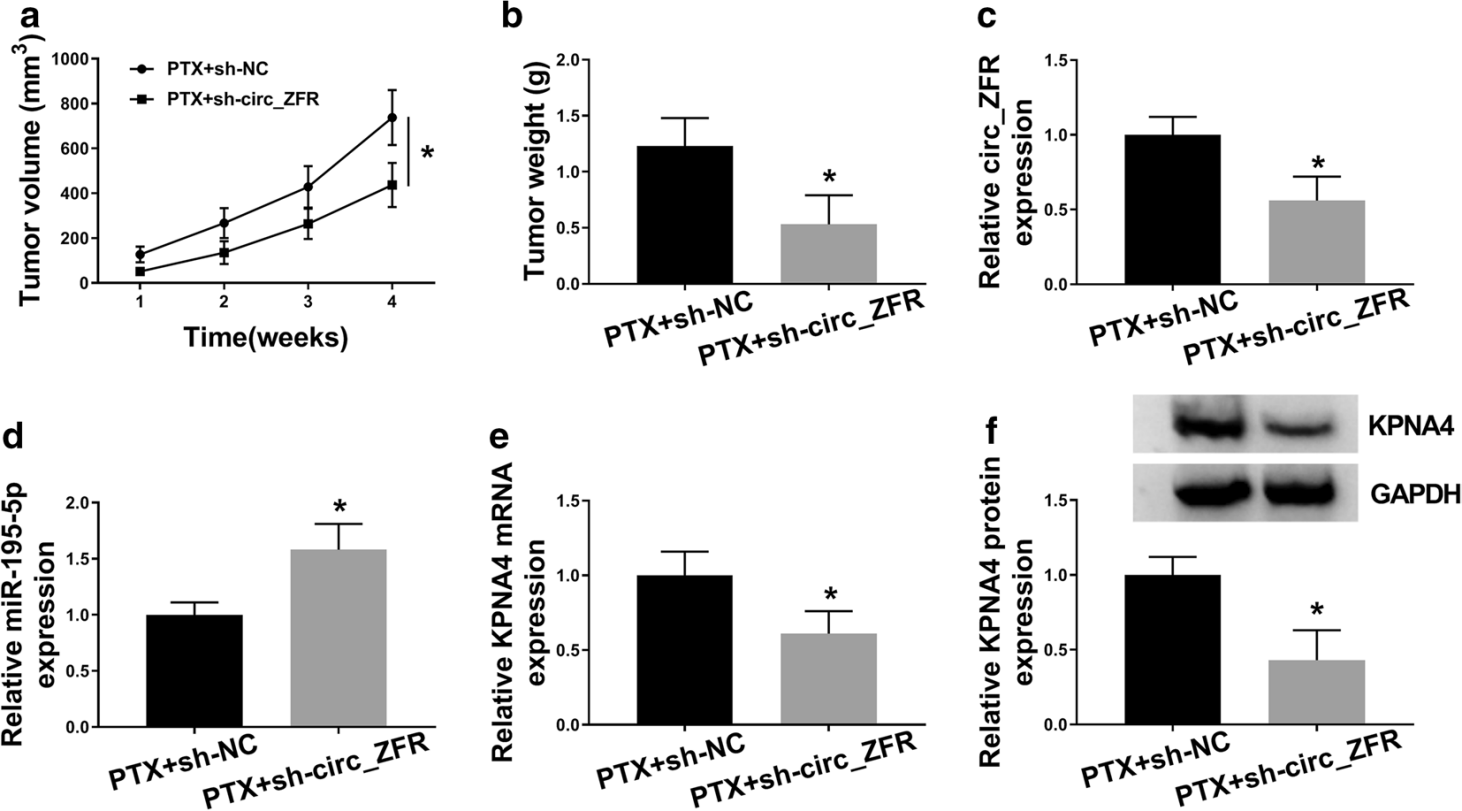
Silencing of circ_ZFR suppressed PTX resistance of NSCLC in vivo. **a** Tumor volume was estimated every week. **b** Tumor weight was measured after 4 weeks. **c**–**e** The levels of circ_ZFR, miR-195-5p and KPNA4 mRNA in the harvested tumors were determined by qRT-PCR assay. **f** The protein level of KPNA4 in the harvested tumors was measured by western blot analysis. *P < 0.05

In order to elucidate the function of circ_ZFR in PTX resistance of NSCLC in vivo. A549/PTX cells stably transfected with sh-circ_ZFR or sh-NC were inoculated into the nude mice. After 1 week, the mice were intraperitoneally administrated with 3 mg/kg PTX or equal volume of PBS every week. As a result, xenograft mice treated with sh-circ_ZFR showed a marked decrease in tumor volume and weight compared to sh-NC groups (Fig. 8a, b). Moreover, we found that the levels of circ_ZFR, KPNA4 mRNA and KPNA4 protein were reduced and the level of miR-195-5p was raised in the tumor tissues obtained from PTX + sh-circ_ZFR groups compared to PTX + sh-NC groups (Fig. 8c–f). These data indicated that circ_ZFR knockdown contributed to PTX sensitivity of NSCLC in vivo.

## Discussion

Chemoresistance is a big challenge for the clinical therapy of human cancers, including NSCLC. With the progression of high throughput sequencing technology, diverse circRNAs have been verified to participate in regulating the development of tumorigenesis and chemoresistance. Our goal of the present research was to determine the roles of circ_ZFR in regulating the malignant phenotypes and PTX sensitivity of NSCLC. As a result, circ_ZFR facilitated PTX resistance, cell proliferation and metastasis and repressed cell cycle arrest and apoptosis in PTX-resistant NSCLC cells by regulation of miR-195-5p/KPNA4 axis.

In NSCLC, the essential roles of circRNAs in tumor progression and drug resistance have gradually attracted the attention of researchers. For instance, circ_0002483 was lowly expressed in NSCLC cell lines and its elevation restrained cell invasion and migration and improved taxol sensitivity in NSCLC cells by sponging miR-182-5p [11]. Circ_0076305 level was raised in DDP-resistant NSCLC cells and circ_0076305 deficiency reduced DDP resistance via regulating miR-296-5p/STAT2 axis [12]. These outcomes suggested that circRNAs played different roles in drug resistance of NSCLC. As for circ_ZFR, Zhang et al. disclosed that circ_ZFR was enhanced in NSCLC tissues and cells, and promoted NSCLC cell growth and metastasis by modulation of miR-101-3p/CUL4B axis [26]. Li et al. unraveled that circ_0072083 interference repressed DDP resistance, cell colony formation, cell cycle and metastasis and facilitated apoptosis in NSCLC cells by regulating miR-545-3p/CBLL1 axis [15]. Herein, high level of circ_ZFR was detected in chemoresistant NSCLC tissues and cells. Circ_ZFR deficiency enhanced PTX sensitivity, accelerated cell apoptosis and cell cycle arrest and restrained cell proliferation and motility in PTX-resistant NSCLC cells in vitro. Moreover, circ_ZFR knockdown repressed the resistance of NSCLC to PTX in vivo. Collectively, circ_ZFR contributed to cell progression and PTX resistance in NSCLC.

For mechanism analysis, circ_ZFR was identified to serve as the sponge for miR-195-5p and then elevated KPNA4 expression. Luo et al. declared that miR-195-5p upregulation restrained cell growth, cell cycle and facilitated apoptosis in NSCLC cells via binding to CEP55 [27]. Zheng et al. demonstrated the tumor suppressive role of miR-195-5p in NSCLC by regulating CIAPIN1 [28]. Yu et al. reported that miR-195-5p directly targeted TUBB to represses the resistance of NSCLC cells to microtubule-targeting agents [21]. Xiong et al. suggested that miR-195-5p participated in regulating the glycolysis and growth of NSCLC through circMYLK/miR-195-5p/GLUT3 axis [29]. All these findings indicated that miR-195-5p played crucial roles in the development and chemoresistance of NSCLC. Herein, our results showed that miR-195-5p was declined in PTX-resistant NSCLC tissues and cells. Suppression of miR-195-5p could effectively restore the impacts of circ_ZFR knockdown on PTX sensitivity and cell progression in PTX-resistant NSCLC cells. Moreover, overexpression of miR-195-5p improved PTX sensitivity, cell cycle arrest and apoptosis and inhibited proliferation and metastasis in PTX-resistant cells, whereas these impacts could be abolished by elevating KPNA4. Our results indicated that miR-195-5p suppressed PTX resistance and cell progression in PTX-resistant NSCLC cells by targeting KPNA4. In support of our results, Wang et al. declared that KPNA4 aggravated NSCLC progression via acting as the target of miR-33a-5p [22]. Of note, although KPNA4 has been demonstrated to act as the target of diverse miRNAs, such as miR-340-5p [30], miR-3619-5p [31] and miR-567 [32], we demonstrated the interaction between miR-195-5p and KPNA4 in chemoresistance of NSCLC for the first time. However, there are still some limitations in this study. For example, the sample size was not sufficient and we did not directly investigate the functions of KPNA4 in NSCLC development and chemoresistance.

**Fig. 9:**
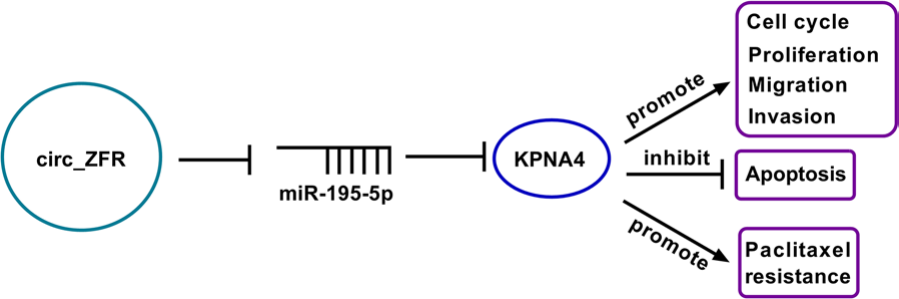
The schematic diagram of circ_ZFR in the regulation of PTX resistance and NSCLC development

In summary, our findings indicated that circ_ZFR promoted cell cycle process, proliferation, migration and invasion, suppressed apoptosis and enhanced PTX resistance in NSCLC by modulating miR-195-5p/KPNA4 axis (Fig. 9), which might offer novel targets to overcome the resistance of NSCLC to PTX.

## Supplementary Information

**Additional file 1: Figure S1.** The effects of circ_ZFR, miR-195-5p and KPNA4 on the levels of CyclinD1, Bcl-2 and Bax in PTX-resistant NSCLC cells. (A-C) The protein levels of CyclinD1, Bcl-2 and Bax in A549/PTX and H460/PTX cells transfected with si-NC or si-circ_ZFR were measured by western blot assay. (D-F) The protein levels of CyclinD1, Bcl-2 and Bax in A549/PTX and H460/PTX cells transfected with si-NC, si-circ_ZFR, si-circ_ZFR + anti-miR-NC or si-circ_ZFR + anti-miR-195-5p were measured by western blot assay. (G-I) The protein levels of CyclinD1, Bcl-2 and Bax in A549/PTX and H460/PTX cells transfected with miR-NC, miR-195-5p, miR-195-5p + pcDNA or miR-195-5p + KPNA4 were measured by western blot assay. *P < 0.05.

**Additional file 2: Figure S2.** CircZFR negatively regulated KPNA4 expression. (A and B) After A549/PTX and H460/PTX cells were transfected with si-NC, si-circ_ZFR, si-circ_ZFR + pcDNA or si-circ_ZFR + KPNA4, the mRNA and protein levels of KPNA4 were detected by qRT-PCR assay and western blot assay, respectively. *P < 0.05.

## Data Availability

Please contact the correspondence author for the data request.
