# Supplementary figures and images for: Circ_ZFR contributes to the paclitaxel resistance and progression of non-small cell lung cancer by upregulating KPNA4 through sponging miR-195-5p
Source: Cancer Cell Int. 2021 Jan 6;21:15. doi: 10.1186/s12935-020-01702-0 (PMC7788748; doi:10.1186/s12935-020-01702-0)

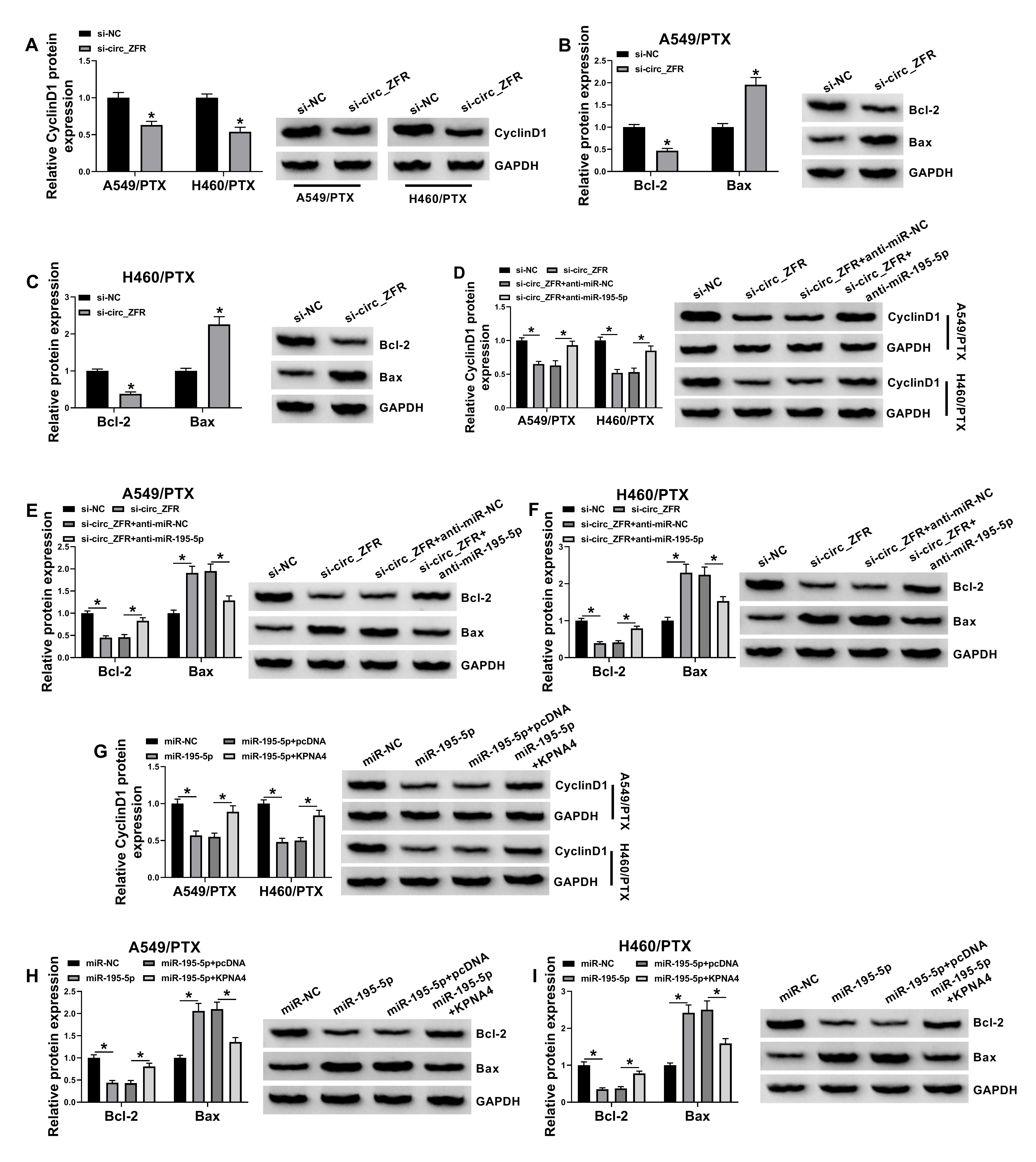

Supplement: Supplementary file 1 — Additional file 1: Figure S1. The effects of circ_ZFR, miR-195-5p and KPNA4 on the levels of CyclinD1, Bcl-2 and Bax in PTX-resistant NSCLC cells. (A-C) The protein levels of CyclinD1, Bcl-2 and Bax in A549/PTX and H460/PTX cells transfected with si-NC or si-circ_ZFR were measured by western blot assay. (D-F) The protein levels of CyclinD1, Bcl-2 and Bax in A549/PTX and H460/PTX cells transfected with si-NC, si-circ_ZFR, si-circ_ZFR + anti-miR-NC or si-circ_ZFR + anti-miR-195-5p were measured by western blot assay. (G-I) The protein levels of CyclinD1, Bcl-2 and Bax in A549/PTX and H460/PTX cells transfected with miR-NC, miR-195-5p, miR-195-5p + pcDNA or miR-195-5p + KPNA4 were measured by western blot assay. *P < 0.05. [file 12935_2020_1702_MOESM1_ESM.tif]

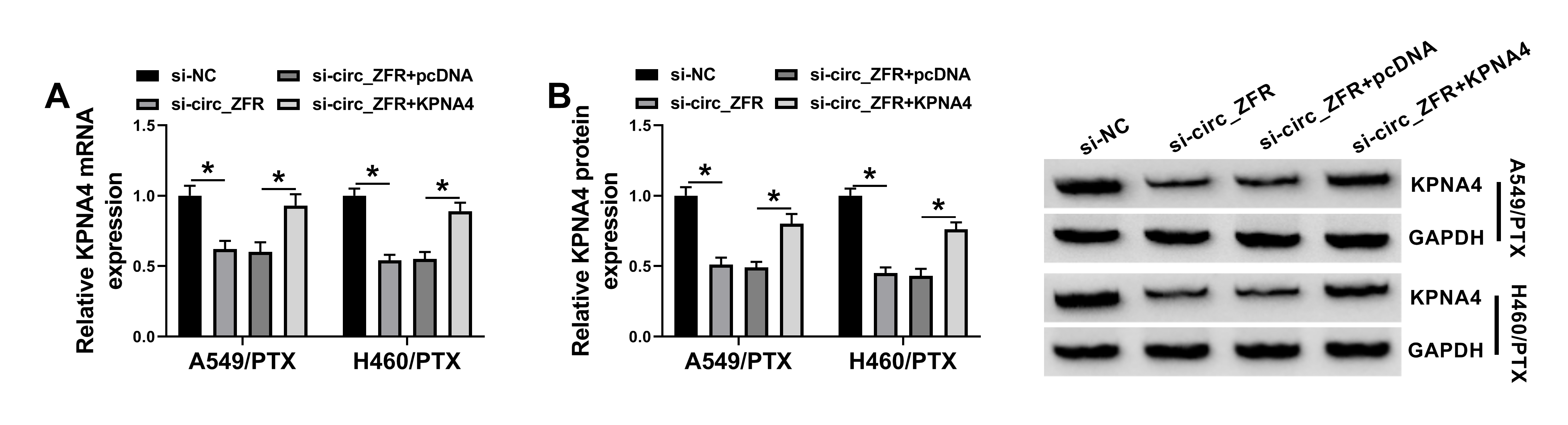

Supplement: Supplementary file 2 — Additional file 2: Figure S2. CircZFR negatively regulated KPNA4 expression. (A and B) After A549/PTX and H460/PTX cells were transfected with si-NC, si-circ_ZFR, si-circ_ZFR + pcDNA or si-circ_ZFR + KPNA4, the mRNA and protein levels of KPNA4 were detected by qRT-PCR assay and western blot assay, respectively. *P < 0.05. [file 12935_2020_1702_MOESM2_ESM.tif]
